# Tiam1 as a Signaling Mediator of Nerve Growth Factor-Dependent Neurite Outgrowth

**DOI:** 10.1371/journal.pone.0009647

**Published:** 2010-03-19

**Authors:** Shahrzad Shirazi Fard, Julianna Kele, Marçal Vilar, Gustavo Paratcha, Fernanda Ledda

**Affiliations:** 1 Laboratory of Molecular and Cellular Neuroscience, Department of Neuroscience, Karolinska Institute, Stockholm, Sweden; 2 Division of Molecular and Cellular Neuroscience, Institute of Cellular Biology and Neuroscience Prof. Dr. E. De Robertis (IBCN)-CONICET, School of Medicine, University of Buenos Aires, Buenos Aires, Argentina; 3 Departament de Bioquimica i Biología Molecular, Universitat de Valencia, Valencia, Spain; of Minnesota, United States of America

## Abstract

Nerve Growth Factor (NGF)-induced neuronal differentiation requires the activation of members of the Rho family of small GTPases. However, the molecular mechanisms through which NGF regulates cytoskeletal changes and neurite outgrowth are not totally understood. In this work, we identify the Rac1-specific guanine exchange factor (GEF) Tiam1 as a novel mediator of NGF/TrkA-dependent neurite elongation. In particular, we report that knockdown of Tiam1 causes a significant reduction in Rac1 activity and neurite outgrowth induced by NGF. Physical interaction between Tiam1 and active Ras (Ras-GTP), but not tyrosine phosphorylation of Tiam1, plays a central role in Rac1 activation by NGF. In addition, our findings indicate that Ras is required to associate Tiam1 with Rac1 and promote Rac1 activation upon NGF stimulation. Taken together, these findings define a novel molecular mechanism through which Tiam1 mediates TrkA signaling and neurite outgrowth induced by NGF.

## Introduction

The neurotrophin family, including NGF, BDNF, NT-3/4, is the best characterized group of growth factors that influence nervous system development. Neurotrophins acting through their specific tyrosine kinase receptors (RTKs), TrkA (for NGF), TrkB (for BDNF and NT-4) and TrkC (for NT-3) have been involved in multiple biological processes, including survival, migration and neurite outgrowth of various neuronal populations. Once activated, the RTKs trigger intracellular signal transduction cascades, including those mediated by Ras/MAPK and PI3 kinase (PI3K) pathways [1], [2], [3]. Although different neurotrophins activate common signaling pathways through their respective RTKs, differences exist among them [4].

Rho-like GTPases, including Rho, Rac1 and Cdc42 are critical proteins in transducing neurotrophin signals to the actin cytoskeleton. While, activation of Rac1 promotes actin polymerization inducing axonal growth, RhoA activation inhibits the outgrowth. In particular, it has been described that neurite outgrowth induced by NGF and TrkA is mediated by the activation of Rac1, which results in the inactivation of RhoA [5], [6].

As molecular switches, Rho GTPases cycle between an inactive GDP-bound state and an active GTP-bound state [7]. Guanine nucleotide exchange factors (GEFs) stimulate the exchange of GDP for GTP to generate the activated form [8]. In contrast, GTPases activating proteins (GAPs), increase the intrinsic rate of GTP hydrolysis inactivating the Rho GTPases [9]. Thus, the activation state of the Rho GTPases depends on the balance of activities of GEFs and GAPs.

While certain GEFs can activate several Rho GTPases, other GEFs are specific for each Rho GTPase [10], [11]. The T-lymphoma invasion and metastasis 1 (Tiam1) is a Rac1-specific GEF that is highly expressed in the developing brain [12], [13]. The Tiam1 protein is 1591 amino acids long and contains several distinct domains [14]. In particular the presence of plekstrin homology (PH) and Ras-binding domains (RBD) makes Tiam1 a good candidate for linking PI3K and/or Ras to Rac1 in response to NGF, functioning as a mediator of neuronal differentiation.

Many evidences support a role of Tiam1 in membrane ruffling, neuronal cell spreading, neurite formation, and dendritic spine morphogenesis in a Rac-dependent manner [15], [16], [17], [18]. Recent studies have revealed that the Rac1-GEF Tiam1 complex plays a critical role in NT-3 and BDNF-mediated signal transduction leading to actin cytoskeletal remodeling that is essential for Schwann cell migration and neurite outgrowth of cortical neurons, respectively [19], [20]. Although activation of Trk receptors triggers common intracellular signaling pathways, involving Rac1, differences in the mechanism of activation of the Rac1-exchange factor Tiam1 between both TrkB and TrkC have been reported. While Tiam1 appear to mediate NT-3 and BDNF-induced Rac1 activation, it is not clear whether activation of Rac1 by Tiam1 is critical for biological responses induced by NGF and TrkA.

Finally, the prominent expression of Tiam1 in NGF-responsive neurons, such as developing sensory and sympathetic neurons as well as the neuronal cell line PC12, led us to consider a possible role of Tiam1 in NGF-induced TrkA downstream signaling and neurite extension.

## Results

### Tiam1 is required for Rac1 activation induced by NGF

**Figure 1:**
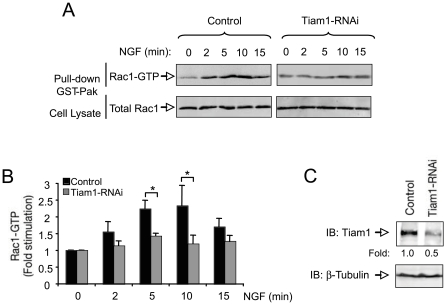
Tiam1 is required for Rac1 activation induced by NGF. A) PC12 cells were transfected with control or pSuper-Tiam1 RNAi (Tiam1 RNAi). After stimulation with NGF (50 ng/ml), Rac1 activation was assessed by αPak-GBD pull-down assay, followed by immunoblot (IB) with anti-Rac1 antibodies. The bottom panel shows total Rac1 present in cell lysates. B) The histogram shows the quantification of Rac1 activity after NGF stimulation in PC12 cells transfected with control or pSuper-Tiam1 RNAi. Results are presented as average ± SD from three or four independent experiments. * p<0.05 (Student's t test). C) Endogenous levels of Tiam1 protein were analyzed by immunoblot (IB) of PC12 cells transfected with control or pSuper-Tiam1 RNAi. Numbers below the lanes indicate fold changes relative to cells transfected with control plasmid normalized to the levels of β-Tubulin.

To investigate whether Tiam1 plays a role in Rac1 activation induced by NGF, we knockdown Tiam1 expression in PC12 cells by using the plasmid-based pSuper RNA interference (RNAi) system [21]. It has already been demonstrated that this construct inhibits Tiam1 expression without affecting the levels of other neuronal proteins [22]. To asses the role of Tiam1 in NGF-dependent Rac1 activation, we transfected PC12 cells with control or pSuper-Tiam1 RNAi and the cells were stimulated with NGF for the indicated times (see Figure 1A). The level of active Rac1 (Rac-GTP) was measured by using an affinity-precipitation assay with the GST-tagged Rac1-GTP interacting binding domain of αPak [19], [23], [24]. As it has been previously described, Rac1 was activated in a time-dependent manner after NGF stimulation of PC12 cells. However, the activity of Rac1 was significantly reduced after knocking down Tiam1 expression, indicating that Tiam1 mediates Rac1 activation triggered by NGF (Figure 1A and 1B). Expression of endogenous Tiam1 was markedly down regulated by transfection with pSuper-Tiam1 RNAi, whereas the expression of β-tubulin was unaffected as revealed by immunoblotting (Figure 1C).

### RNAi knockdown of Tiam1 expression inhibits neurite outgrowth of PC12 cells in response to NGF

**Figure 2:**
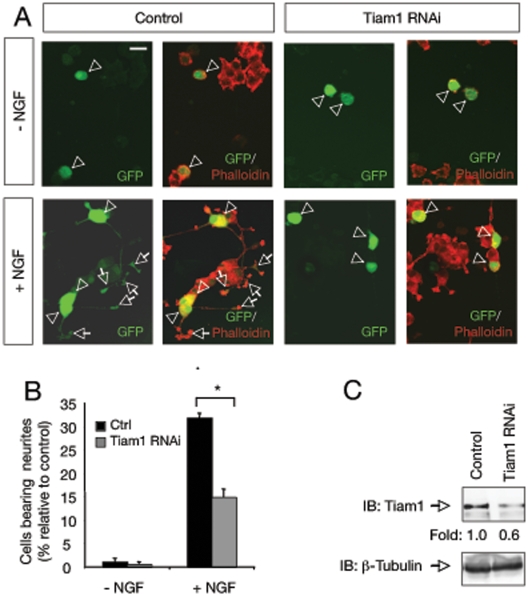
RNAi knockdown of Tiam1 expression inhibits neuronal differentiation of PC12 cells in response to NGF. A) Photomicrographs show PC12 cells transfected with control or pSuper-Tiam1 RNAi (Tiam1 RNAi) together with a GFP expression vector. After 72 h of NGF treatment, the cells were fixed and stained with phalloidin. Arrowheads indicate neuronal cell bodies and arrows denote neurite tips. Scale bar: 10 µm. B) The histogram shows the quantification of the relative number of GFP positive PC12 cells bearing neurites longer than 1.5 cell body diameters after 72 h of treatment with NGF. The results are presented as average ± SD of a representative experiment performed in triplicate. *p<0.001 (ANOVA followed by Student Newman Keuls). The experiment was repeated three times with similar results. C) Endogenous levels of protein were analyzed by immunoblot (IB) in PC12 cells transfected with control or pSuper-Tiam1 RNAi.

As it is known that Rac1 is involved in the NGF-induced actin cytoskeletal remodeling required for neurite elongation, we next investigated the possible role of Tiam1 in this process. To examine whether Tiam1 plays a role in NGF-induced neuronal differentiation, PC12 cells were transfected with pSuper-Tiam1 RNAi or control plasmid in combination with an enhanced green fluorescent protein (GFP) expression vector. Cells were maintained in the presence of NGF for 72 h and stained with phalloidin, which revealed the polymerized actin filaments (Figure 2A). To exclude the possibility that the effects seen with Tiam1 RNAi were due to apoptosis, the morphology of the nuclei was assessed using the nuclear staining DAPI or To-Pro3. Interestingly, knockdown of Tiam1 inhibited NGF-induced PC12 neurite outgrowth. A significant reduction (≈50%, p<0.001) in cells bearing neurites was observed after transfection with pSuper-Tiam1 RNAi (Figure 2A and 2B). This result demonstrates that Tiam1 is involved in NGF-mediated morphological differentiation of PC12 neuronal cells. Downregulation of endogenous Tiam1 in PC12 cells was controlled by western blot (Figure 2C).

### Tiam1 is expressed in SCG neurons and mediates neurite outgrowth of sympathetic neurons in response to NGF

**Figure 3:**
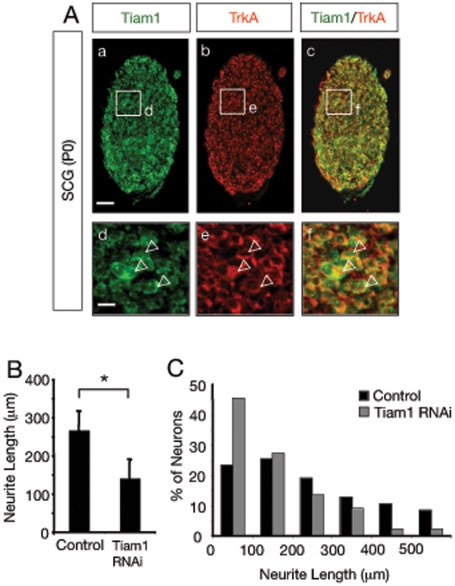
Tiam1 is expressed in SCG neurons and mediates neurite outgrowth in response to NGF. A) Colocalization of Tiam1 and TrkA in SCG sections from P0 mice detected by immunofluorescence. d-f, are higher magnification images of the boxes contained in a-c respectively. Arrowheads indicate individual cells showing Tiam1-TrkA colocalization. Scale bars a-c, 30 µm; d-f, 12.5 µm. B) Histogram showing the inhibition of neurite outgrowth in SCG neurons by knockdown of Tiam1 expression. Dissociated SCG neurons were transfected with control or pSuper-Tiam1 RNAi together with a GFP expression vector and cultured with NGF. After 36 h in culture, the neurons were fixed and stained with anti-β-Tubulin antibodies. The results are shown as the average ± SD of a representative experiment performed in triplicate *p<0.05 (Student's t test). The experiment was repeated three times with similar results. C) The histogram shows the distribution of neurons carrying neurites classified in different length categories after transfection with GFP in the absence (control) or in the presence of pSuper-Tiam1 RNAi. A total of 47 control neurons and 45 Tiam1 RNAi-transfected neurons from a representative assay were evaluated. Note the shift to the left in the distribution of neurons that received the pSuper-Tiam1 RNAi construct.

To determine whether Tiam1 could play an *in vivo* role in NGF signaling, we examined weather Tiam1 and the NGF receptor, TrkA, are co-expressed in superior cervical ganglion (SCG) and dorsal root ganglion (DRG) neurons, two neuronal populations responsive to NGF. Transverse sections of SCG and DRG as well as primary dissociated SCG neurons were assessed by immunofluorescence using anti-Tiam1 and anti-TrkA antibodies. Staining of dissociated neurons and tissue sections obtained from P0 mice revealed a striking co-localization of Tiam1 and TrkA expression in SCG and DRG neurons (Figures 3A and S1). This data suggests that Tiam1 may play a physiological role in NGF/TrkA-driven DRG and SCG neuron development.

We also examined the role of Tiam1 in the neurite outgrowth induced by NGF in SCG neurons. To this end, dissociated SCG (P0) neurons were transfected with control or pSuper-Tiam1 RNAi constructs together with a GFP expression vector. In agreement with the results obtained in PC12 cells, the neurite outgrowth stimulated by NGF was significantly reduced in cells transfected with pSuper-Tiam1 RNAi (neurite length ± SD in µm, control: 267.6±48.9; pSuper-Tiam1 RNAi: 146.0±49.2; *p<0.05) (Figure 3B and 3C). The shiftment to the left in the distribution of neurons that received the pSuper-Tiam1 RNAi, suggests that Tiam1 is involved in NGF-mediated neurite elongation more than in the initial steps of neurite formation (Figure 3C). Survival of GFP-positive neurons transfected with control or pSuper-Tiam1 RNAi vector was also evaluated. No differences were observed in NGF-promoted neuron survival between the two groups. Data expressed as average values ± SD are as follow: Control: 92.5±1.8; pSuper-Tiam1 RNAi: 90.6±3.1, n = 3; p>0.05).

### NGF-dependent Tiam1 activation is mediated by Ras

Recent advances made in the understanding of the mechanisms through which Tiam1 is activated by the neurotrophins BDNF and NT3, revealed that this GEF could be regulated at two different levels: (i) binding to the BDNF receptor, TrkB, and subsequent Tiam1 activation by tyrosine phosphorylation and (ii) activation of Tiam1 by a NT3/TrkC- induced mechanism involving the direct protein-protein interaction between Ras-GTP and the Tiam1 RBD domain [19], [20].

**Figure 4:**
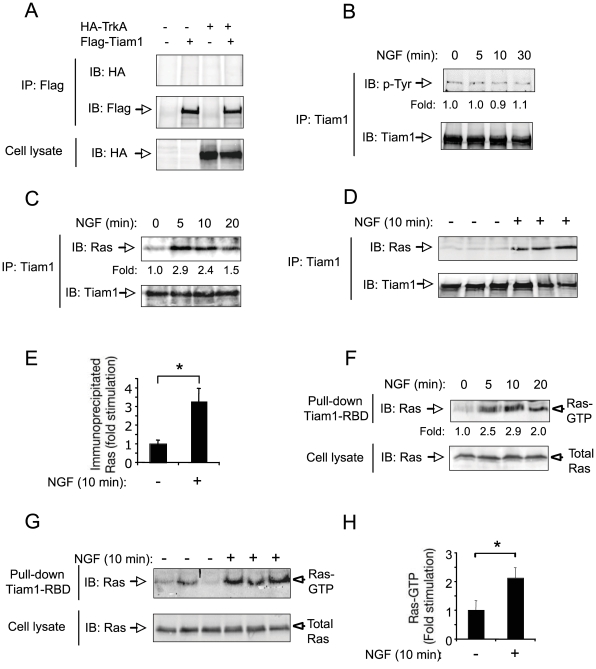
NGF-induced Tiam1 activation is mediated by Ras, and independent of tyrosine phosphorylation. A) Tiam1 does not interact with TrkA. COS cells were transfected with Flag-tagged Tiam1 (C1199) and HA-tagged TrkA and cell extracts were analyzed by immunoprecipitation (IP) with anti-Flag antibodies followed by immunoblot (IB) with antibodies against HA. Reprobing of the same blot with anti-Flag antibodies and anti-Tiam1 are shown below. The bottom panel shows HA-tagged TrkA expression in total cell lysates. The experiment was repeated three times with identical results. B) Tiam1 is not tyrosine phsophorylated after NGF stimulation. PC12 cells treated with NGF at the indicated time points were immunoprecipitated (IP) with anti-Tiam1 antibodies followed by immunoblot (IB) with antibodies against phosphotyrosine (P-Tyr). Reprobing of the same blot with anti-Tiam1 antibodies is shown. Fold change normalized to the levels of Tiam1 is indicated. The experiment was repeated three times with similar results. C-E) Interaction between endogenous Tiam1 and Ras in PC12 cells treated with NGF. Analysis was done by immunoprecipitation (IP) with anti-Tiam1 antibodies followed by immunoblot (IB) with anti-Ras antibodies. The bottom panels show endogenous Tiam1 expression in total PC12 extracts. (C) Kinetic of Tiam1-Ras interaction at different time points after NGF stimulation. Fold change normalized to the levels of Tiam1 is indicated. (D), Tiam1-Ras interaction measured after 10 minutes (min) of NGF treatment in three independent experiments. (E), The histogram shows the quantification of Tiam1-Ras interaction measured in D. Results are presented as average ± SD from three independent experiments. *p<0.05 (Student's t test). F-H) Ras activity measured by pull-down assay using GST-Tiam1 (720-841) in cell lysates prepared from PC12 treated with NGF. The bottom panels show total Ras measured in cell lysates. (F) Kinetic of Ras activity at different time points after NGF stimulation. Fold change normalized to the levels of Ras in the total lysates is indicated. (G) Ras activity measured after 10 min of NGF treatment in three independent experiments. (H) The histogram shows the quantification of Ras activity measured in G. Results are presented as average ± SD from three independent experiments. *p<0.05 (Student's t test).

To investigate whether Tiam1 interacts with the NGF receptor, TrkA, we performed a co-immunoprecipitation assay in COS cells transfected with hemagglutinin (HA)-tagged TrkA receptors in the absence or presence of Flag-tagged Tiam1. Figures 4A and S2B show that TrkA could not be specifically co-immunoprecipitated with Tiam1 in COS cells. Furthermore, no physical interaction between endogenous TrkA and Tiam1 was detected in PC12 cells treated with NGF (Figure S2A). In contrast, we were able to detect endogenous association between Tiam1/NMDA receptor subunit NR2B, and Tiam1/BDNF receptor TrkB, two molecular interactions previously detected by co-immunoprecipitation from rat brain synaptosomes (Figure S2C) [20], [22].

Since tyrosine phosphorylation has previously been reported to modulate Tiam1 GEF activity by BDNF [20], we evaluated the level of Tiam1 tyrosine phosphorylation in PC12 cells treated with NGF at different time-points. Tiam1 phosphorylation was determined by immunoprecipitation followed by immunoblotting with anti-phosphotyrosine antibodies. Using this method, we did not detect changes in the tyrosine phosphorylation level of Tiam1 in response to TrkA activation by NGF (Figure 4B).

Interestingly, Tiam1 can also be activated by direct interaction with the neurotrophin effector H-Ras [19]. Consistent with this, a recent study indicated that Ras binding to the Ras-binding domain (RBD) of Tiam1 plays a central role in Tiam1 activation induced by neurotrophin-3 (NT-3)/TrkC [19]. Thus, in order to explore the contribution of Ras to Tiam1 activation triggered by NGF, we performed co-immunoprecipitation assays between Ras and Tiam1. Association between endogenous Tiam1 and Ras was observed in PC12 treated with NGF in a time-dependent manner. Figure 4C-4E shows a significant (* p<0.001) association between Ras and Tiam1 after 10 min of NGF stimulation. This result suggests that Tiam1 physically associates with active Ras (Ras-GTP) through its RBD domain. In order to confirm this, we used a GST-tagged Tiam1 fusion protein containing the RBD (aa 720-841, Tiam1-RBD), which has the ability to specifically associate with Ras-GTP but not with Ras-GDP [19]. Thus, we performed a pull-down assay using the Tiam1-RBD on PC12 cells extracts treated or not with NGF at different time-points. In agreement with the NGF-induced association between endogenous Tiam1 and Ras, a robust increase in the amount of Ras-GTP precipitated with Tiam1-RBD was detected after NGF treatment, with a peak within the first 10 min (Figure 4F). Quantification and statistical analysis performed at 10 min after stimulation, revealed a significant (* p<0.05) increase in the association between active Ras and Tiam1 (Figure 4G and 4H).

### Ras is required for Tiam1-Rac1 association and Rac1 activation in response to NGF

**Figure 5:**
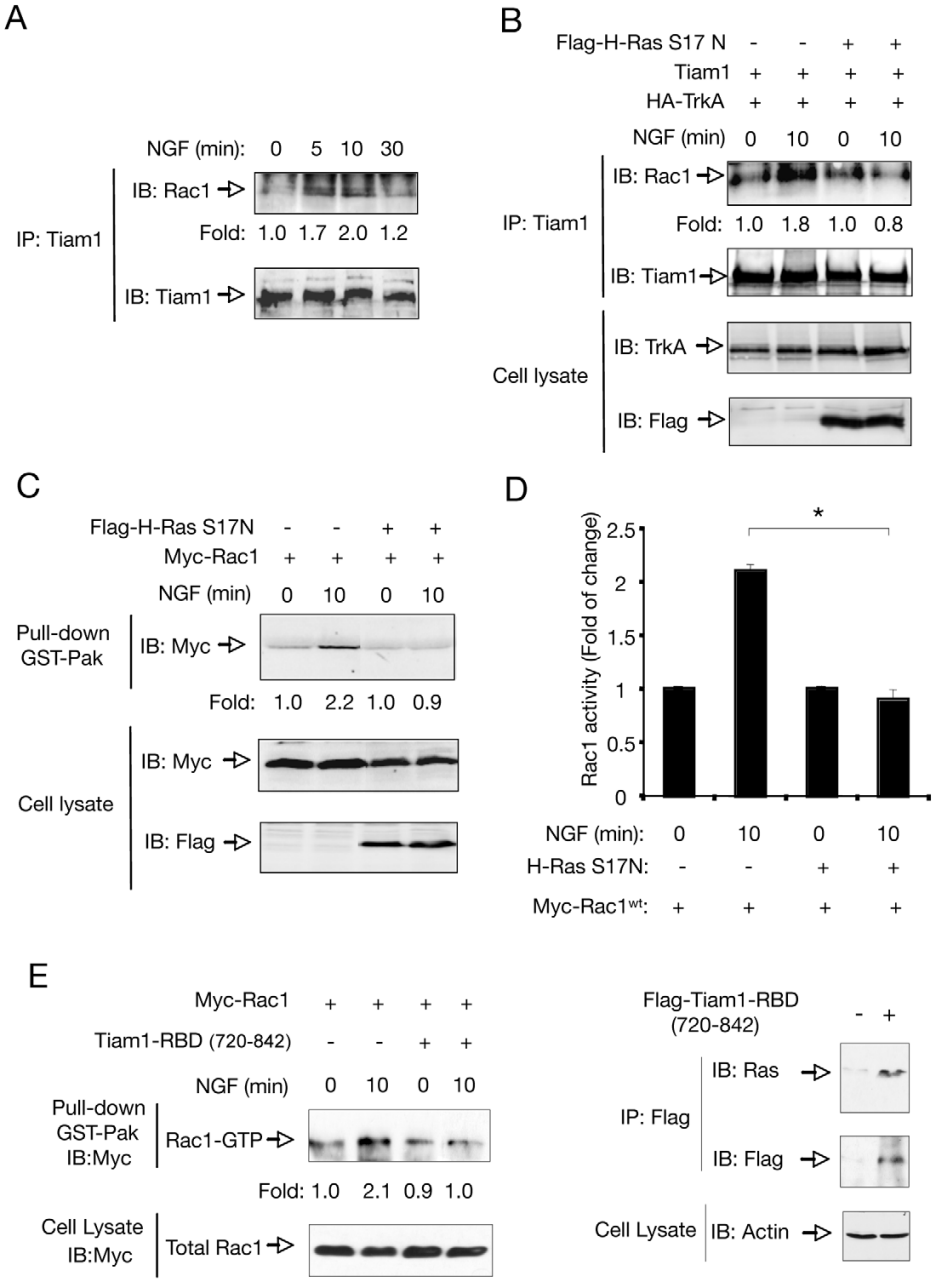
H-Ras is required for NGF-induced Tiam1-Rac1 interaction and Rac1 activation. A) Co-immunoprecipitation between Tiam1 and Rac1 in response to NGF. The panel shows an increase in the amount of endogenous Rac1 precipitated with Tiam1 at different time points. Cell lysates were immunoprecipitated (IP) with anti-Tiam1 antibodies followed by immunoblot (IB) with antibodies against Rac1. Reprobing of the same blot with anti-Tiam1 antibodies is shown. Fold change normalized to the levels of Tiam1 is indicated. The experiment was repeated three times with similar results. B) COS cells were transfected with Tiam1, HA-tagged TrkA (HA-TrkA) and control or Flag-tagged dominant-negative (DN) H-Ras (Flag-H-Ras S17N) expression vectors. After NGF stimulation, the interaction between Rac1 and Tiam1 was assayed by immunoprecipitation (IP) with anti-Tiam1 antibodies followed by immunoblot (IB) with anti-Rac1 antibodies. Reprobing of the same blot with Tiam1 antibodies is shown below. Fold change normalized to the levels of Tiam1 is indicated. The figure also shows TrkA and Flag-H-Ras S17N expression in total cell extracts. The experiment was repeated two times with similar results. C) PC12 cells were transfected with Myc-tagged Rac1 (Myc-Rac1) and control or Flag-H-Ras S17N expression vectors. After stimulation with NGF, the activity of Rac1 was evaluated by αPak-PBD pull-down assay, followed by immunoblot (IB) with anti-Myc antibodies. Fold change of Rac1 activity is indicated. The figure also shows total Myc-Rac1 and Flag-H-Ras S17N expression in cell lysates. The experiment was repeated three times with similar results. D) The histogram shows the quantification of Rac1 activity measured in C. Results are presented as average ± SD from three independent experiments. *p<0.0001 (Student's t test). E) In the left panel, Hek 293-TrkA cells were transfected with Myc-Rac1 in the presence or in the absence of Flag-tagged Tiam1-RBD (aa 720-842). After 10 min stimulation with NGF, the activity of Rac1 was evaluated by αPak-PBD pull-down assay, followed by immunoblot (IB) with anti-Myc antibodies. The figure also shows total Myc-Rac1 expression in cell lysates. The experiment was repeated two times with similar results. Right panel, to confirm the expression and functionality of the Flag-Tiam-RBD (aa 720-842), of Hek 293-TrkA were transfected or not with this construct. After 10 min stimulation with NGF, the cell lysates were immunoprecipitated with anti-Flag followed by IB with anti-Ras and anti-Flag antibodies.

Then, we examined the ability of Tiam1 to interact physically with Rac1 in response to NGF. To investigate Tiam1-Rac1 interaction, we performed co-immunoprecipitation assays between endogenous Tiam1 and Rac1 proteins in PC12 cells treated with NGF at different time-points. Previous work has demonstrated that endogenous Rac1 associated to Tiam1 can be specifically co-precipitated with anti-Tiam1 antibodies [25]. In our system, activation of Tiam1 was evidenced by a substantial increase (1.7- to 2.0-fold) in the amount of Rac1 coupled to Tiam1 in PC12 cells treated with NGF for 5-10 min (Figure 5A), revealing that the two proteins are able to interact when expressed at physiological levels. It is known that Tiam1, like other GEFs, may preferentially interact with the GDP-bound form of Rac1, convert it to the GTP-bound form and then dissociate from the GTP-bound form to allow its interaction with downstream effectors [19]. Thus, our data suggests that the interaction between Tiam1 and Rac1 detected here after NGF treatment should involve the GDP-bound form or nucleotide-free form of Rac1 proteins.

To further investigate the mechanistic details underlying Tiam1-mediated Rac1 activation by NGF, we analyzed whether H-Ras was required for both Tiam1-Rac1 interaction and Rac1 activity in response to NGF. To this purpose, we performed a co-immunoprecipitation assay in COS cells transfected with TrkA and Tiam1 constructs in the absence or in the presence of dominant-negative (DN) H-Ras (H-Ras S17N). Expression of DN-H-Ras construct blocked NGF-induced Tiam1-Rac1 interaction, evidenced by an inhibition in the amount of Tiam1 co-precipitated with Rac1 (Figure 5B). Likewise, transfection of PC12 cells with dominant-negative H-Ras inhibited Rac1 activation induced by NGF (Figures 5C and 5D, and S4).

To further confirm that Tiam1-RBD mediates NGF-induced Rac1 activation, we used a fragment of Tiam1 containing the amino acids 720-842 (Tiam1-RBD) to elicit dominant- negative effects [19]. Transfection of Tiam1 (aa 720-842) inhibited NGF-induced Rac1 activation in Hek 293-TrkA cells (Figure 5E, left panel).

Taken together, these findings indicate that H-Ras is required to promote the physical association between Tiam1 and Rac1 and subsequent Rac1 activation in response to NGF.

## Discussion

**Figure 6:**
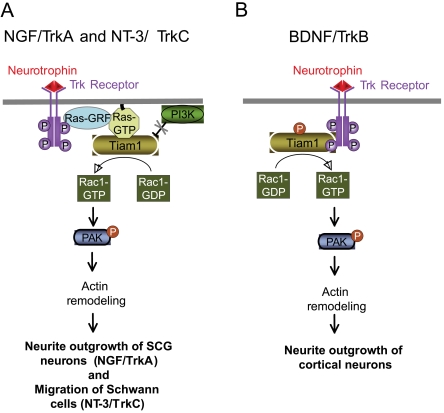
Model depicting the role of Tiam1 in neurotrophin-induced Trk receptor signaling and biology. Panels A and B show the two main mechanisms through which the GEF Tiam1 is activated by neurotrophins. A) Activation of the GEF Tiam1 by NGF/TrkA and NT-3/TrkC requires a physical interaction between Ras-GTP and Tiam1 through its Ras-binding domain. B) On the other hand, TrkB binds and tyrosine-phosphorylates Tiam1 upon BDNF stimulation.

NGF promotes neuronal differentiation through the activation of the members of the Rho family of small GTPases, RhoG, Rac1 and Cdc42. However, the molecular mechanisms regulating the cytoeskeletal changes necessary for neurite outgrowth are not totally understood. In this work, we identify the Rac1-GEF Tiam1 as an important mediator of NGF/TrkA-dependent neurite outgrowth. We show that knockdown of Tiam1 expression with siRNA causes a significant reduction in both Rac1 and neurite outgrowth activity induced by NGF stimulation. In addition, interaction between Tiam1 and active Ras, but not tyrosine phosphorylation of Tiam1, plays a central role in the activation of Rac1 by NGF. Taken together, our results demonstrate that NGF and its signaling receptor TrkA promote neurite outgrowth and Rac1 activity enhancing the activation of Tiam1 by direct interaction with the small GTPase H-Ras. On the basis of these and previous findings, we summarize the two mechanisms used by neurotrophins/Trk receptors to activate the Rac1-specific GEF activator, Tiam1. Thus, while BDNF acting through TrkB receptor directly binds and activates Tiam1 by a delayed tyrosine phosphorylation (around 30 min after stimulation) [20], NGF and NT3, acting through their respective receptors, TrkA and TrkC, activate Tiam1 through a faster mechanism (peaking around 5-10 min) that involves the direct interaction between Ras-GTP and the Tiam1 RBD domain [19] (Figure 6). In agreement with this, Tiam1-dependent Rac1 activation induced by BDNF is also more delayed (peaking at 45 min) than the Rac1 activity detected after NT3 and NGF stimulation. In the particular case of NGF, we additionally demonstrate that there is not a direct interaction between its receptor, TrkA, and Tiam1 and that the activation of Tiam1 does not require tyrosine phosphorylation (Figure 4A and 4B). However, since all Trk receptors activate Ras, the issue of why TrkB-induced Tiam1 activation does not require Ras activity, arise. Although the cytoplasmic domains of TrkA and TrkB receptors are highly similar, a discrete sequence that accounts for functional differences between both receptors has been detected [3], [4]. Interestingly, this evidence opens the possibility that TrkA and TrkB activate Tiam1 acting through different intracellular signaling mechanisms. The fact that TrkB promotes a delayed activation of Tiam1 by phosphorylation does not rule out the possibility that this same receptor could also induce a faster Ras-dependent Tiam1 activation similar to the previously reported for TrkC [19] and to the one showed here for TrkA. Thus, the role of Ras on TrkB-mediated Tiam1 activation also requires further investigation.

Previous studies have shown that in PC12 cells, constitutively active PI3K is involved in neurite outgrowth. This effect could be reverted by dominant negative Rac1 [26], suggesting that PI3K activity is influencing Rac1 function. However, extension and maintenance of neurites provoked by PI3K is incomplete, indicating that PI3K activity is not sufficient to induce full differentiation of PC12 cells [26], [27]. Consistent with this, Nusser et al (2002) described that only partial inhibition of Rac1 activity can be observed by pharmacological inhibition of PI3K induced by NGF [6]. As Tiam1 is able to bind specifically to the lipid products of PI3K through its PH domains, it might mediate GTP loading of Rac1 after PI3K activation by NGF [14], [28], [29], [30], [31]. However, and consistent with recent studies, we find that the formation of the signaling complex Tiam1-Rac1 is not abrogated by inhibition of PI3K using Ly294002, indicating that Tiam1 mediates Rac1 activation by a PI3K-independent mechanism [32], [33] (Figure S3).

Although our findings provide evidence showing that Tiam1 cooperates with Ras to mediate NGF-induced Rac1 activation, the residual activity of Rac1 observed in cells treated with Tiam1 RNAi in the presence of NGF, suggests the existence of alternative Tiam1-independent mechanisms through which NGF can activate Rac1. Other mechanisms implicated in Rac1 activation by NGF have been reported: the Rac1-GEF P-Rex1, involved in cell migration [34], and the ternary complex RhoG-Elmo-Dock180 [35], involved in neuronal differentiation. Why would multiple Rac1 activators be required to regulate NGF-dependent biological functions? It is possible that these GEFs or complexes of activation have different tissue or subcellular distributions or are expressed at different developmental periods or time-points after NGF stimulation. In fact, it has been described that the expression of RhoG and its GEF-Trio are strongly induced in PC12 cells several hours after NGF stimulation [5], [36]. In addition, these GEFs might confer specificity, regulating different downstream signaling pathways and thereby controlling biological outputs. Interestingly, the Rac1-GEF P-Rex1 has been clearly involved in NGF-induced PC12 cell migration [34], but its role promoting neuronal differentiation is still controversial [37]. In addition, the GEFs Vav2/3, which activate a broad range of Rho GTPases, have also been involved in the formation of short processes leading to neurite outgrowth induced by NGF [38]. Depletion of Vav2/3 partially inhibited early NGF-dependent activation of Rac1/Cdc42, indicating that additional GEFs, such as Tiam1, could be involved in this biochemical event. Likewise, several GEFs, including Sos1, Sos2, Vav2, Tiam1, pRex1 and Asef [34], [39], [40], have been shown to transduce the growth signal from EGF receptor to Rac1, revealing a high degree of intracellular complexity to regulate Rac1 activation in response to trophic factors.

Our findings indicate that by modulating Tiam1 function, NGF and TrkA receptors can induce Rac1-dependent signaling cascades, resulting in cytoskeletal remodeling required for neurite outgrowth. The accurate regulation of the activity of Rho GTPases is crucial for the normal development of the nervous system. In the developing nervous system, neurite outgrowth is an essential process underlying the formation of highly specific pattern of neuronal connections [41], [42]. Abnormalities in neurite formation and extension have been speculated to cause disorganization of neuronal network and eventual neural diseases. Thus, abnormal signaling through Rho family GTPases is an important cause of mental retardation that is associated with impairments in neuronal morphology, connectivity and synaptic plasticity [43], [44]. Therefore, further characterization of the mechanisms by which Tiam1 and other Rho-specific regulatory proteins are controlled in neurons may contribute to understand the underlying causes of mental retardation and other neurological disorders.

## Materials and Methods

### Ethics Statement

Animal experiments were approved by Stockholms Norra djurförsöksetiska nämnd (Dnr: 360/06).

### Cell lines and recombinant proteins

COS and Hek 293-TrkA cells were grown in DMEM supplemented with 10% fetal bovine serum (FBS). PC12 cells were grown in DMEM supplemented with 5% horse serum and 10% FBS. NGF was purchased from Promega. GST-tagged Tiam1 (720-841) and αPak-CRIB were purified using E.coli BL21, as previously described [19], [45].

### Plasmids and Cell Transfections

Transient transfection of COS cells was performed using the calcium phosphate method, and cells were harvested 48 h later. PC12 cells were transfected using Fugene-6 (Roche) following manufacturer's instructions. Primary superior cervical ganglion (SCG) neurons were transfected by nucleofection using an Amaxa device. Briefly, dissociated neurons were suspended in 50 µl of Amaxa buffer with 3 µg of total plasmid DNA (0.5 µg of GFP +2.5 µg of pSuper-Tiam1 RNAi or control vector). Suspended cells were then transfected to the nuclofection cuvettes and nucleofected using the Amaxa device following the manufacturer's protocol.

Plasmids encoding C1199 Tiam1 (aa 393-1590) and the pSuper-Tiam1 RNAi were kindly provided by Dr. Michael Greemberg (Harvard Medical School, Boston, USA) [46] [22]. This sequence was not homologous to Tiam2, a guanine nucleotide exchange factor-GEF- with significant identity to Tiam1, or any other known gene as determined by a BLAST search [22] and was not able to interfere the expression of Tiam2 (data not shown). Plasmid encoding GFP was obtained from Clontech. The Ras binding domain of Tiam1 (Tiam1-RBD: aa 720-842) was amplified by PCR and cloned in the EcoRI/SmaI sites of the pflag-CMV2 vector. The plasmids encoding pCMV-Flag-tagged-H-RasS17N (GDP-bound form) and the GST-tagged expression vector pET42a containing the Tiam1-Ras binding domain (Tiam1-RBD, aa 720-841) were generously provided by Dr. Junji Yamauchi (Department of Pharmacology, National Research Institute for Child Health and Development, Tokyo, Japan) and Dr. Eric M. Shooter (Department of Neurobiology, Standford University School of Medicine, Stanford, USA). The plasmid encoding the GST-tagged GTPase binding domain (GBD) of PAK (PAK-GBD) was kindly provided by Dr. Hollis T. Cline (Cold Spring Harbor Laboratory, Cold Spring Harbor, NY, USA).

### Sympathetic neuron cultures

Superior cervical ganglion (SCG) neurons, from embryonic day 21 (E21) or P0 Sprague Dawley rats (Scanbur, Karlsunde, Denmark) were prepared as previously described [47]. Briefly, the ganglia were dissociated with trypsin and collagenase, and seeded onto poly-ornithine and laminin coated plates. The neurons were maintained in DMEM:F12 medium containing 10% FBS and AraC (10 µM) and supplemented with NGF (50 ng/ml).

### Immunoprecipitation and Western blotting

Cells were lysated at 4°C in buffer containing 0.5% Triton X-100, 1% β-octylglucoside plus protease and phosophatase inhibitors. Protein lysates were clarified by centrifugation and analyzed by immunoprecipitation and Western blotting as previously described [48]. Depending on the experiment, the samples were run in a discontinuos (8-15% SDS-PAGE) gel in order to be able to detect, in the same blot, proteins with different molecular weights. The blots were scanned in a Storm 860 FluorImage (Molecular Dynamics), and quantifications were done with ImageQuant software (Molecular Dynamics). Numbers below the lanes indicate fold of induction relative to control normalized to total levels of the target protein.

The antibodies were obtained from various sources as follows: anti-phosphotyrosine (p-Tyr) and anti-Tiam1 were from Santa Cruz Biotechnology, anti-Ras was from Upstate, anti-Rac1 and anti-TrkB were from BD Biosciences Pharmingen, anti-Flag antibodies were from Sigma.

### Pull down assays for Rac1 and Ras activation

To detect active, GTP-bound Rac1, we performed the pull-down assay using GST-αPak-CRIB, which specifically binds to Rac1 GTP-bound forms. The band intensity in the immunoblot was quantified, and the levels of Rac-GTP were normalized to the amount of each total GTPase.

To detect active, GTP-bound Ras, we performed the pull-down assay using GST-Tiam1 (720-841), which specifically binds to the GTP-bound form. The band intensity in the immunoblot was quantified, and the levels of Ras-GTP were normalized to the amount of total Ras.

### Immunofluorescence

Cryostat sections of E15.5 and newborn (postnatal day 0, P0) mice were blocked with 10% donkey serum and incubated with polyclonal anti-TrkA (dilution 1/100, R&D systems), and anti-Tiam1 (dilution 1/100, Santa Cruz). Second antibodies were from Jackson ImmunoResearch. Photographs were obtained using a Zeiss LSM510 confocal microscope.

### Neurite outgrowth assays

For PC12 cell differentiation assays, the cells were transfected with GFP or GFP plus pSuper-Tiam1 RNAi using Fugene-6 reagent in complete medium. The next day the cells were plated on poly-D-lysine coated coverslips in 24 multi well plates and cultured in 1% serum containing medium supplemented with NGF (50 ng/ml). After 72 h, the cells were fixed with 4% paraformaldheyde (PFA) and stained with Rhodamine-conjugated phalloidin. After staining, confocal microscopy was performed in a Zeiss confocal microscope. The number of cells bearing neurites longer than 1.5 cell bodies was quantified relative to the total number of neurons counted in at least 10 random fields of three different wells in each experiment. PC12 cell differentiation was evaluated in three independent experiments.

Neurite outgrowth assays were performed in dissociated cultures of E21 rat SCG neurons. Primary cultures were prepared as previously described (see above). Neurons were transfected with GFP or GFP plus pSuper-Tiam1 RNAi by nucleofection and cultured in the presence of NGF (50 ng/ml) for 36 h. Then the cells were fixed with 4% PFA and stained with anti-β-tubulin to identify neuronal cells. Neuronal survival was evaluated using the nuclear stain 4′, 6′ –diamino-2-phenylindole (DAPI) (Invitrogen) or To-Pro3 (Molecular Probes). GFP-positive neurons containing fragmented or condensed nuclear staining were scored as apoptotic cells and not computed in the differentiation assays. Quantification of neurite length was done with a Zeiss LSM510 confocal microscope using the Axiovision software (Zeiss) version 2.01.

## Supporting Information

Figure S1 Tiam1 expression in DRG and SCG neurons. (A–F) Colocalization of Tiam1 and TrkA in DRG sections from E15.5 mice detected by immunofluorescence. (D–F), are higher magnification images of the boxes contained in (A–C), respectively. Scale bars (A–C) 25 µm; (D–F) 12.5 µm. (G–L) Colocalization of Tiam1 and TrkA in SCG dissociated cells obtained from P0 rat detected by immunofluorescence. (J–L), are higher magnification images of the boxes contained in (G–I) respectively. Scale bars (G–I) 12 µm; (J–L) 5 µm.
(3.73 MB TIF)

Figure S2 Tiam1 does not interact with TrkA receptors. A) PC12 cells were stimulated with NGF and cell extracts were analyzed by immunoprecipitation with anti-Tiam1 antibodies followed by immunoblot with antibodies against TrkA. Reprobing of the same blot with anti-Tiam1 antibodies is shown below. The figure also shows MAPK activation as a control of ligand stimulation. B) COS cells were transfected with Flag-tagged Tiam1 (C1199) and HA-tagged TrkA and cell extracts were analyzed by immunoprecipitation (IP) with anti-Flag antibodies followed by immunoblot (IB) with antibodies against HA. Reprobing of the same blot with anti-Flag antibodies and anti-Tiam1 are shown below. The bottom panel shows HA-tagged TrkA expression in total cell lysates. The experiment was repeated three times with identical results. C) Control experiment showing the interaction between Tiam1/NR2B and Tiam1/TrkB in rat brain synaptosomes. Lysate from rat synaptosomes was immunoprecipitated with anti-Tiam1 antibodies or with control antibodies and then immunoblotted with anti-NR2B and anti-TrkB antibodies. The expression of NR2B, TrkB and Tiam1 was confirmed in synaptosome total lysates (Lys) by immunoblotting. Reprobing of the same blot with anti-Flag antibodies and anti-Tiam1 are shown below. The bottom panel shows HA-tagged TrkA expression in total cell lysates. The experiment was repeated three times with identical results. C) Control experiment showing the interaction between Tiam1 and NR2B in rat brain synaptosomes. Lysate from rat synaptosomes was immunoprecipitated with anti-Tiam1 antibodies or with control antibodies and then immunoblotted with anti-NR2B antibodies. The expression of NR2B and Tiam1 was confirmed in synaptosome total lysates (Lys) by immunoblotting.
(3.43 MB EPS)

Figure S3 NGF-promoted Tiam1-Rac interaction is independent of PI3K activity. A) Tiam1-Rac1 interaction induced by NGF treatment (10 min) is not abrogated in the presence of the PI3K inhibitor Ly294002 (50 µM). Cell lysates were immunoprecipitated (IP) with anti-Tiam1 antibodies followed by immunoblot (IB) with antibodies against Rac1. Reprobing of the same blot with anti-Tiam1 antibodies is shown. Fold change normalized to the levels of Tiam1 is indicated. The experiment was repeated two times with similar results. B) Control of phospho-Akt inhibition by the PI3K antagonist Ly294002. Phospho-Akt (P-Akt) induced by NGF treatment (10 min, 50 ng/ml) is abrogated in the presence of the PI3K inhibitor Ly294002 (50 µM). Endogenous levels of P-Akt were analyzed by immunoblot (IB) of PC12 cell extracts. The bottom panel shows the total level of β-tubulin presents in cell lysates.
(0.51 MB EPS)

Figure S4 Ras is required for NGF-induced Rac1 activation in PC12 cells. A and B) The panels show two additional experiments indicating the requirement of H-Ras for NGF-induced Rac1 activation. PC12 cells were transfected with Myc-tagged Rac1 (Myc-Rac1) and control or Flag-H-Ras S17N (Ras dominant negative) expression vectors. After stimulation with NGF (50 ng/ml), the activity of Rac1 was evaluated by αPak-PBD pull-down assay, followed by immunoblot (IB) with anti-Myc antibodies. Fold of change of Rac1 activity is indicated. The figure also shows total Myc-Rac1 and Flag-H-Ras S17N expression in cell lysates. C) Immunoblot control showing the exogenous expression of eppitope tagged Rac1 in COS cells transfected with empty vector or Myc tagged-Rac1 construct.
(0.65 MB EPS)
