# Supplementary figures and images for: Tiam1 as a Signaling Mediator of Nerve Growth Factor-Dependent Neurite Outgrowth
Source: PLoS One. 2010 Mar 19;5(3):e9647. doi: 10.1371/journal.pone.0009647 (PMC2841637; doi:10.1371/journal.pone.0009647)

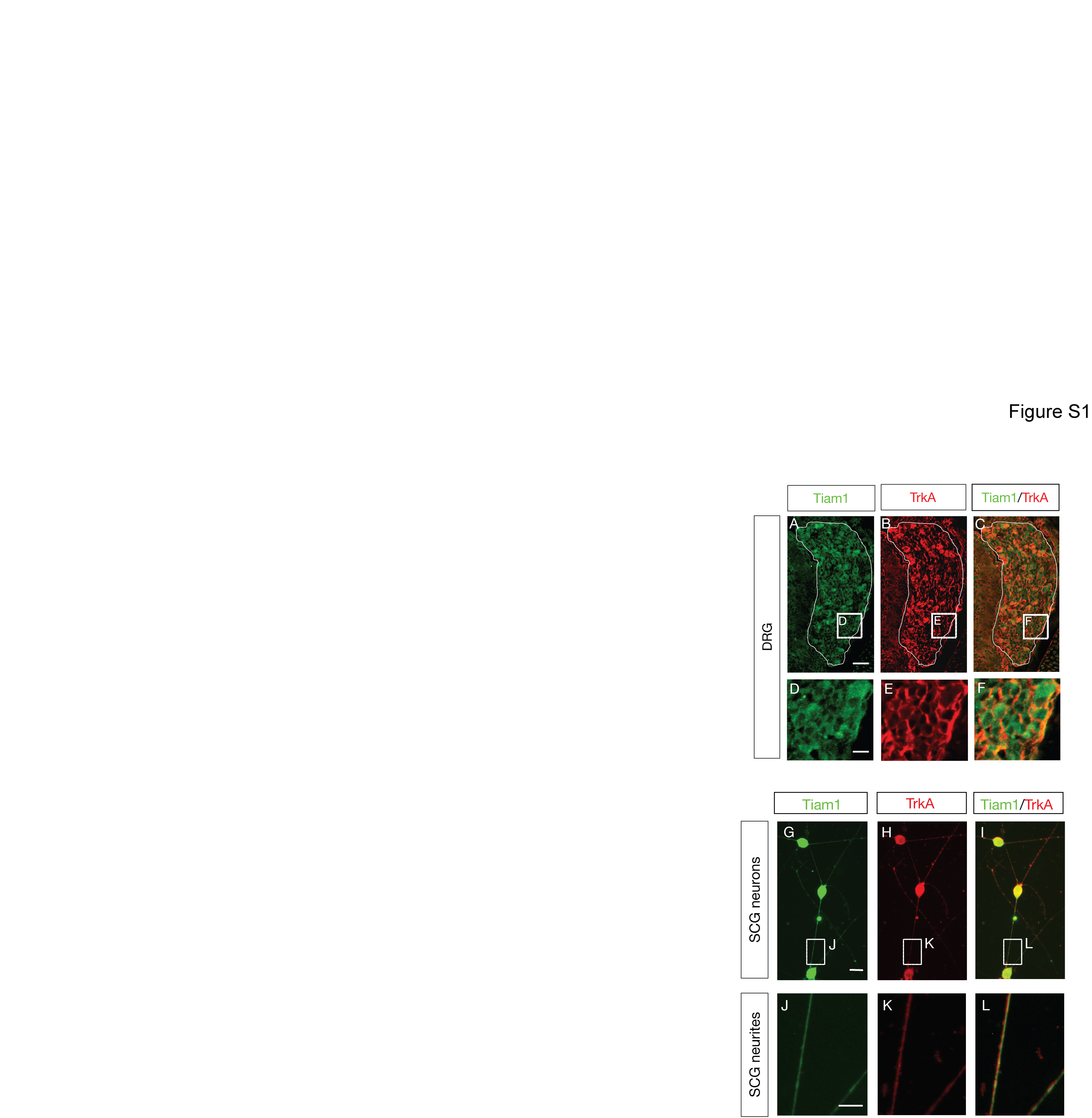

Supplement: Figure S1 — Tiam1 expression in DRG and SCG neurons. (A–F) Colocalization of Tiam1 and TrkA in DRG sections from E15.5 mice detected by immunofluorescence. (D–F), are higher magnification images of the boxes contained in (A–C), respectively. Scale bars (A–C) 25 µm; (D–F) 12.5 µm. (G–L) Colocalization of Tiam1 and TrkA in SCG dissociated cells obtained from P0 rat detected by immunofluorescence. (J–L), are higher magnification images of the boxes contained in (G–I) respectively. Scale bars (G–I) 12 µm; (J–L) 5 µm. (3.73 MB TIF) [file pone.0009647.s001.tif]
